# The Mode of Inheritance of Scheuermann's Disease

**DOI:** 10.1155/2013/973716

**Published:** 2013-09-12

**Authors:** A. M. Zaidman, M. N. Zaidman, E. L. Strokova, A. V. Korel, E. V. Kalashnikova, T. V. Rusova, M. V. Mikhailovsky

**Affiliations:** ^1^Institute of Traumatology and Orthopaedics, Frunze Street 17, Novosibirsk 630091, Russia; ^2^Rambam Health Care Campus, 6 Ha'Aliya Street POB 9602, 31096 Haifa, Israel

## Abstract

The mode of Scheuermann's disease inheritance and its phenotypic traits in probands and their relatives were studied in 90 pedigrees (90 probands and 385 relatives). The disorder was identified as a genetically related pathology inherited by autosomal dominant type, controlled by a mutant major gene, as a kyphotic deformity without signs of vertebral bodies' anomaly and torsion. Morphological and biochemical studies showed disturbance in the structure of vertebral growth plate anterior aspects at the level of deformity, defects in proliferation and differentiation of chondrocytes, and change in proteoglycan spectrum in cells and matrix. Twelve candidate genes were studied in chondrocytes isolated from vertebral growth plates of patients with Scheuermann's disease. The study results included disorder in the IHH gene expression and preservation of the expression of PAX1, two aggrecan isoforms, link protein, types I and II collagen, lumican, versican, growth hormone and growth factor receptor genes, and proliferation gene. Preservation of the SOX9 gene (transcription gene) probably indicates posttranscriptional genetic disorders. The study is under way.

## 1. Introduction

The juvenile kyphotic deformity of the spine has been known since antiquity but was identified by Scheuermann as a nosological entity only in 1921. Scheuermann argued that this pathology was largely associated with aseptic necrosis of the ossification centers of the ring apophyses. This theory was further supported by Jones and Wise [1] and Parsch G [2] which supposed this pathology was basically associated with ischemic disorders of ossification centers. However, no morphological evidence for impaired blood circulation in the forming vertebral bodies has been found. The hypothesis that Scheuermann's disease (SD) is caused by initial changes in the intervertebral disc [3–5] and vertebral osteoporosis [6–8] was rejected. Attention focused on vertebral body endplates. Morphological studies performed by Aufdermaur [9, 10] revealed the presence of sparse disorganized fibrils in the cartilage matrix, changes in the architectonics of collagen fibers, presumably associated with disturbed collagen synthesis. Based on that evidence, Dommisse [11] and Linthoudt and Revel [12] proposed pathogenetic mechanisms for SD, believing that, if cartilage tissue is congenitally compromised, the disc penetration into the vertebral body may result from mechanical loading. More detailed studies were conducted by Ippolito and Ponseti [13] and Ascani et al. [14]. These authors argue that SD results from a compromised architectonics of the cartilage growth plate of vertebral body, a compromised integrity of cells and matrix, and disbalance of proteoglycan/collagen ratio, which, all together, lead to the wedge-shaped deformity of vertebral bodies under mechanical loading. The lack of firm morphological evidence of changes in the structural components of the spine in SD patients is probably the main reason why the opinions on the pathogenesis of this disease differ so much. 

There are reports of aggregations of Scheuermann's disease in families and a number of reports suggesting a hereditary character of this disorder [15–19]. A high concordance for Scheuermann's disease has been demonstrated in monozygotic twins [20]. The analysis of separate pedigrees suggests that Scheuermann's disease has an autosomal dominant inheritance [21, 22].

However, Scheuermann's disease is still generally considered as a hereditary disorder of unknown etiology [23]. An attempt to test the involvement of Duffy, COL1A1, and COL1A2 genes in the determination of Scheuermann's disease in three large pedigrees was unsuccessful [22]. So Scheuermann's disease inheritance remains uncertain, especially as these studies were based on restricted data coming from a few pedigrees. To our knowledge, literature does not contain reports on candidate gene study in chondrocytes of patients—carriers of a putative mutant gene.

Our objective was to study, in correctly ascertained pedigrees, the mode of inheritance and to identify hereditary phenotypic traits of the disorder and candidate genes.

## 2. Materials and Methods

Ninety probands age 9–18 years with Scheuermann's disease and their 385 relatives underwent a clinical genetic examination. They were classified into three groups.

1. Children and adolescents with a verified diagnosis of Scheuermann's disease:
  - 110 individuals (probands and sibs), age 9–18 years,
  - 62 were boys (56.4%) and 48 were girls (43.6%),
  - the male and female ratio was 1.3 : 1,
  - Grade I 12.7%,
  - Grade II—in 66.4%,
  - Grade III—in 20.9%.
2. Adults with a verified diagnosis of Scheuermann's disease:
  - 295 individuals,
  - their age ranged from 21 to 69 years,
  - male and female ratio was 1.36 : 1.
3. Pedigrees of families with Scheuermann's disease. Each pedigree included only one proband:
  - 90 families,
  - thirty-four pedigrees included 1st degree relatives (parents and sibs of the proband),
  - fifty-six pedigrees had a more complex structure—they included 2nd and 3rd degrees relatives (grandmothers, grandfathers, aunts, uncles, and cousins).

In all 385 members of pedigrees have been examined.

All the probands and their relatives with Scheuermann's disease admitted to specialized clinic underwent complete clinical examination including X-ray and MRI and survey by geneticist. The following characteristics were described: sex, age, kyphotic deformation degree and rigidity, and changes of vertebral body structure and shape. The diagnosis of Scheuermann's disease was based on both clinical and radiographic signs. All cases with kyphotic deformity of 25–44° of Cobb angle were classified as Scheuermann's disease Grade I, with 45–65° as Grade II and with 65° and more as Grade III. This classification was made with a regard for progressive structural changes of the spine column tissues.

*Segregation analysis* was performed using MAH-A1 software package. This is a version specially developed to test the monogene diallel hypotheses of alternative trait inheritance [21]. The trait was assumed to be controlled by autosomal diallel gene *A* with genotypes *A*_1_ *A*_1_, *A*_1_ *A*_2_, and *A*_2_ *A*_2_, whose incidence in population is *p* ^2^, 2*pq*, and *q*, respectively (*q* is *A*_2_, allele frequency, *p* = 1 − *q*). The transmission of alleles from parents to offspring is described by transmission probabilities: *τ*(*g*) = Pr(*A*_2_/*g*)—a probability of the parent with genotype *g* to transmit allele *A*_2_ to their offspring. A typical Mendelian gene segregation suggests that *τ*(*g*) = 1, 0.5, and 0 for *g* = *A*_1_ *A*_1_, *A*_1_ *A*_2_, and *A*_2_ *A*_2_, respectively. The alternative trait conditionally assigned as *x* = 0 (a norm) and *x* = 1 (Scheuermann's disease) was analyzed. The phenotype correspondence with genotype is determined by the probability Pr(*x*/*g*), described by the genotype penetrance *w*(*g*): Pr(1/*g*) = *w*(*g*),  Pr(0/*g*) = 1 − *w*(*g*). Genotype penetrance in males [*w*_*m*_(*g*)] can differ from that in females [*w*_*f*_(*g*)]. The monogene hypothesis was tested against the Elston-Stewart test [24]: a hypothesis of monogene control can be accepted if

- a hypothesis of genetic control absence which assumes the equal transmission probabilities (environmental hypothesis) will be rejected;
- hypothesis with Mendelian probabilities and that with transmission probabilities will not differ significantly. Hypotheses were compared using the likelihood ratio test [25].

*Morphohistochemical study* was performed on vertebral growth plates from 25 patients aged 12 to 14 years operated in the Novosibirsk Research Institute of Traumatology and Orthopedics. The same structural components from age-matched children obtained in forensic cases were used as controls. The tissue specimens were fixed in 10% buffered formalin; the bone tissue specimens were decalcified in Trilon B. Sections were deparaffinized and stained with histological (Van Gieson's and Mallory's reactions with hematoxylin-eosin) and histochemical methods (toluidine blue in different pH media, alcian blue, Hale's reaction, and periodic acid Schiff reaction). Controls were performed for all reactions. Redox enzymes in cryostat sections were detected using the Nadi reaction and according to Nachlas. Alkaline and acid phosphatases were detected using the azocoupling method. 

To enable an ultrastructural analysis, the material was fixed in 4% buffered paraformaldehyde, postfixed in 1% OsO_4_, dehydrated with alcohol in increasing concentrations, and embedded in Epon-Araldite. Ultrathin sections were prepared using an LKB Ultratome, counterstained, and examined under a Hitachi-600 electron microscope.

*Biochemical study* included isolation of glycosaminoglycans (GAGs) from cartilage using papain solution in 0.2 M Na-acetate buffer at pH 5.8 with 0.01 M EDTA and 0.01 M cysteine for 18 h at 60°C [26]. Proteins were precipitated by 100% trichloroacetic acid to a final concentration of 5%. The solution was dialyzed against 50 mM Na-acetate buffer at pH 5.0 for 18 h at 4°C. GAGs were precipitated by adding three volumes of 96° ethanol and 4% potassium acetate; the precipitate was dissolved in 0.4 M guanidine chloride with 50 mM sodium acetate at pH 5.8. 

The GAG counts in vertebral body growth plates were derived from hexuronic acid content [27] and sulfated GAG counts [28]. The results are expressed as micrograms of standard matter per milligram of dry tissue weight. The ratio of the GAG counts derived from hexuronic acid content to that derived from sulfated GAG count was considered as the level of sulfation. Chondroitin sulfate C (ICN Biomedicals) was used as the standard.

The GAGs' composition in vertebral body GP was resolved by separation using gel electrophoresis in 1% agarose gel [29] using 50 mM barium acetate buffer at pH 5.0. To eliminate some unwanted GAG species before electrophoresis, the GAGs had been sequentially exposed to chondroitinase AC, chondroitinase ABC, and keratanase in 50 mM Tris-buffer at pH 7.5 for 18 h at 37°C. After electrophoresis, the gel was stained with 0.1% azure in 50 mM sodium formate and 10 mM magnesium chloride at pH 3.5. The stained gel was rinsed with 50 mM acetate buffer at pH 5.8. Chondroitin sulfates A, B, C, keratan sulfate, and heparan sulfate (ICN Biomedicals) were used as markers for GAG identification.

The level of *candidate gene expression* was investigated in cells isolated from vertebral growth plates of four children aged 10 to 14 years with Scheuermann's disease (operational material). Chondroblasts isolated from vertebral growth plates of matched children were used as controls. Hyaline cartilage was washed with Hank's solution containing 1.0 g/liter kanamycin for 15 min, crushed to 1.0–2.0 mm^2^ fragments in a Petri dish with minimum volume of PRMI-1640, placed into siliconized vials with 1.5% collagenase, and incubated for 18–22 h at 37°C on a shaker. The obtained suspension was filtered through Nylon filters and centrifuged at 1500 rpm for 10 min.

*RNA Isolation, RT-PCR, and PCR. *Total RNA was isolated from isolated cells using TRI Reagent (Ambion). RNA was purified from the contamination with genomic DNA by DNA-Free (Ambion) kit. About 2 *μ*g of total RNA, 400 ng of random hexamer (Clontech) primers, and 400 U of M-MLV (Promega) reverse transcriptase and the corresponding buffer were used for one reverse transcription reaction. The reactions without reverse transcriptase were used as a negative control. cDNA synthesis was performed in 40 *μ*L for 60 min at 42°C; then reverse transcriptase was inactivated by heating at 95°C for 10 min. 2 *μ*L aliquots were used for amplification with gene-specific primers. PCR was carried out in 50 *μ*L using 5 U of *Taq *polymerase per reaction in a TRIO-Thermoblock (Biometra) under the following conditions: 3 min at 95°C and 30 cycles of 30 s at 95°C, 30 s at 58°C (SOX9, COL1A1, COL2A1, HAPLN1, GHR, TGFBR, LUM, and IHH), 60°C (AGGRECAN, KLF), or 65°C (PAX1) and 30 s at 72°C. The concentration of MgCl_2_ was 3-4 mM for all reactions. PCR was performed with primers listed in Table 1. PCR for GAPDH with the primers GAPDH-S (5′-TGTTGCCATCAATGACCCCTT-3′) and GAPDH-AS (5′-CTCCACGACGTACTCAGCG-3′) was used as a positive control; PCR mode: 3 min at 95°C and 25 cycles of 20 s at 95°C, 30 s at 58°C, and 15 s 72°C.

## 3. Results and Discussion

Grade I Scheuermann's disease developed most often in children 9–11 years of age. In general, by the age 12–15 most patients had Grade II; by the age 16–18 they had Grade III Scheuermann's disease. The incidence was 1.3 times more frequent in boys than in girls, which is in good agreement with the literature [15, 30]. 

Scheuermann's disease clinically manifests as a kyphotic deformity assessed radiographically by the Cobb method. Patients with Scheuermann's disease Grade I had 25–44° of kyphosis with a curve apex at T7-T8. A kyphosis in Grade II patients amounted 45–65° with an apex also at T7-T8. Grade III curve exceeded 65 degrees, being markedly rigid. All grades showed various radiographic spine abnormalities with various incidences. There was no vertebral body torsion.  Table 2 shows the main radiographical signs typical for each of Scheuermann grades. Grade I is characterized by wedge-shaped vertebral bodies, endplate irregularities, and Schmorl's nodes. Narrowing of intervertebral spaces, vertebral body osteoporosis, and apophysis fragmentation were observed in Grade II. Wedge-shaped vertebral bodies, Schmorl's nodes, intervertebral space narrowing, and vertebral body osteoporosis were observed in Grade III, but the manifestation of endplate irregularities and apophysis fragmentation were less obvious because of the older age of this group. 

The mode of Scheuermann's disease phenotypic trait inheritance was explored in detail in the 78 relatives of probands (45 males and 33 females), age 21–69, with a documented diagnosis (Table 3). 

All members of this group had a marked rigid kyphotic deformity. The curve apex, as in probands, was at the T7-T8 in 80% of cases. Vertebral body changes had an equal incidence in relatives of all three degrees of relation (Table 4).

As in the first group (children and adolescents with Scheuermann's disease), this was associated with narrowing of intervertebral space (92%), but vertebral body osteoporosis was more frequent and prominent (81%), while Schmorl's nodes manifested only in 42% of cases. Endplate irregularities were fewer (3%). There was no evidence of apophysis fragmentation. Such a distribution of radiographic signs is caused by the age structure of the group. It is remarkable that the examined relatives aged 40 and more had shown osteochondrosis (78%), spondylosis (56%), and arthrosis (33%) considered as secondary delayed changes. 

**Figure 1:**
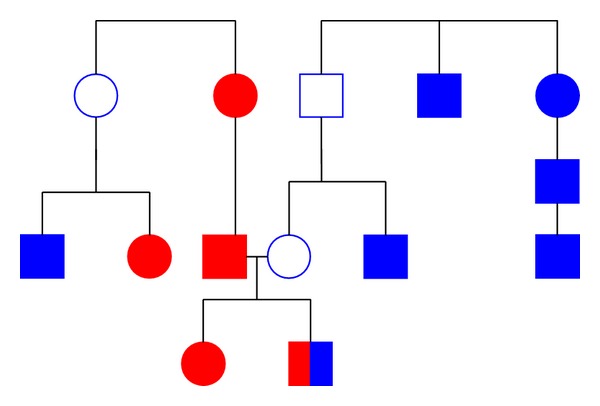
Coexistence of idiopathic scoliosis and Scheuermann's disease in families raises an issue of a single genetic nature of these pathologies: red: idiopathic scoliosis; blue: Scheuermann's disease.

The study of disorder inheritance mode was based on the 88 pedigrees ascertained through a proband with Scheuermann's disease. The analysis has shown that Scheuermann's disease frequency among the closest relatives of the probands was 0.143 (*n* = 21) in sisters, 0.476 (*n* = 21) in brothers, 0.250 (*n* = 84) in mothers, and 0.743 (*n* = 35) in fathers. These values significantly exceed the frequency in general population. They confirm a familial aggregation of Scheuermann's disease. The frequency was higher in brothers and fathers of the probands than in their sisters and mothers. This is consistent with data from [4, 31]. Both parents have been examined in 32 probands. Out of these families five had both parents unaffected, 22 families had an affected father, 4 families had an affected mother, and 1 family had both parents affected. These data confirm that men are more predisposed to Scheuermann's disease than women (Figure 1).

The test of possibility of monogene diallel control of Scheuermann's disease was analysed by segregation analysis.  Table 5 presents test results for different genetic hypotheses. 

The first column contains genetic parameters values for the model assuming that penetrances of all genotypes can take arbitrary values. The second column contains the parameters of dominant model assuming that *w*(*A*_1_ *A*_2_) = *w*(*A*_2_ *A*_2_). These two models do not differ significantly (*x* ^2^ = 0.001). This suggests a dominant mode of Scheuermann's disease inheritance, when a mutant allele is expressed equally in homozygote and heterozygote conditions. The normal genotype penetrance is equal to zero both in males and in females. This means that disease manifestation is possible only in mutant gene carriers, and a mutant gene always manifests in boys, while in girls it manifests in the half of cases.

Thus, the results of clinical and genetic investigation suggest that Scheuermann's disease is a genetically dependent pathology inherited by autosomal dominant type and controlled by a mutant major gene. Deformation manifests during the adolescent growth spurt and progresses from Grade I to Grade III. To ascertain a precise mode of inheritance we made attempts to identify gene candidates having been chosen on the basis of morphological, biochemical, and molecular genetic studies of spine structural components affected by Scheuermann's disease.

### 3.1. Morphological Study of Structural Components of Vertebral Bodies from Patients with Grade III Scheuermann's Disease

**Figure 2:**
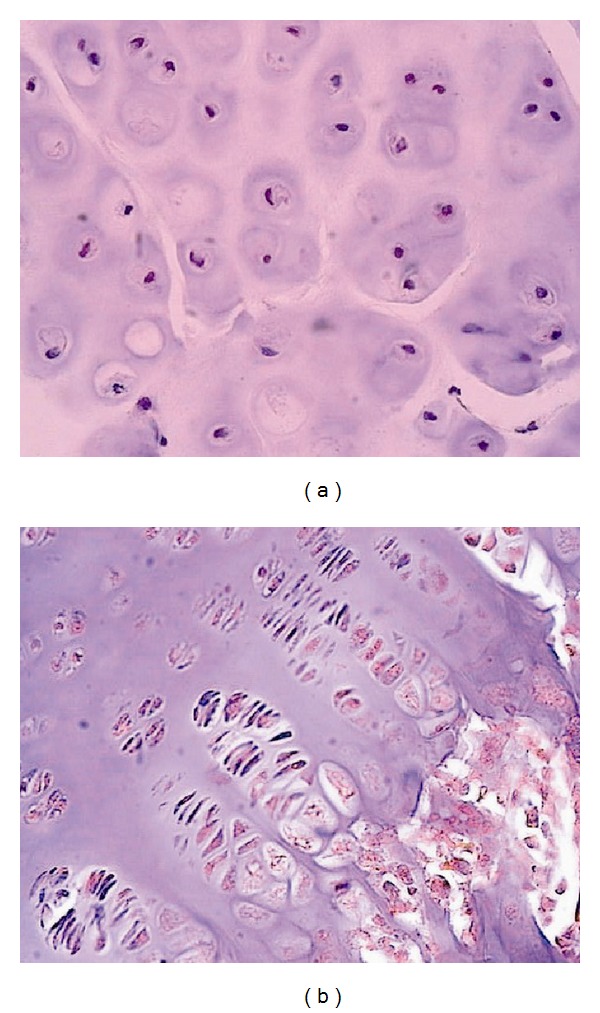
Vertebral growth plate at the level of deformity: (a) cell and matrix disorganization (GP ventral aspects); (b) structural organization of GP is preserved (GP dorsal aspects). Hematoxylin-eosin staining, ×200.

The vertebral body growth plate (at the level of deformity) in the ventral aspects is significantly narrowed and is presented by a degraded, fragmented matrix which reveals disordered arrangement of chondrocytes (Figure 2). 

**Figure 3:**
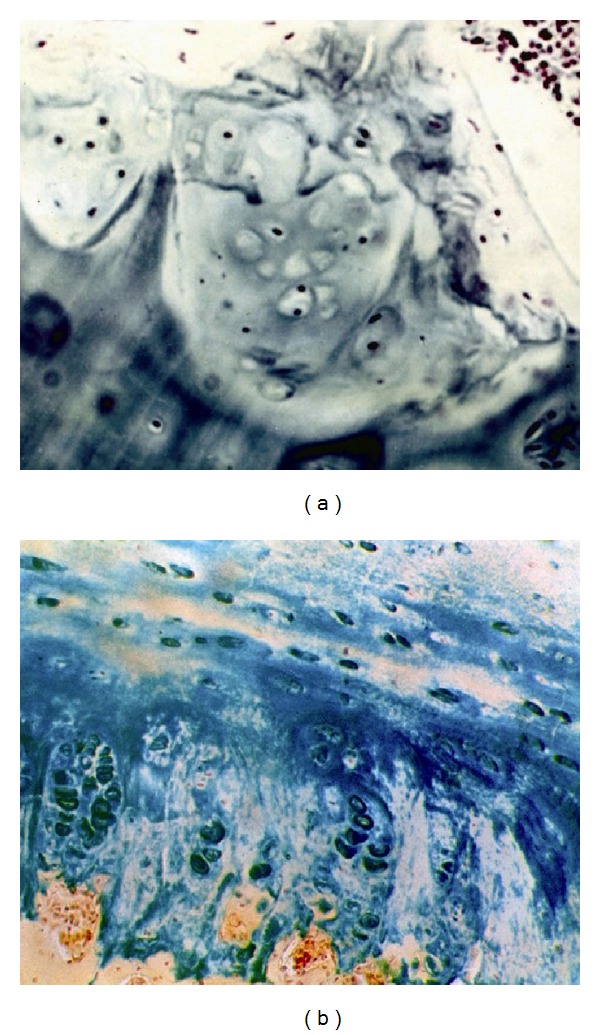
Proteoglycans in cells and matrix of vertebral growth plate at the level of deformity: (a) ventral aspects; (b) dorsal aspects. Hale's reaction, ×200.

Histochemical study revealed trace reaction to low-polymeric chondroitin sulfate (Figure 3(a)) and keratan sulfate. In the dorsal aspects, the structure of vertebral body growth plate is preserved and composed of four layers. Chondroitin sulfates A and C and keratan sulfate in trace amounts are revealed in the matrix and cells (Figure 3(b)).

**Figure 4:**
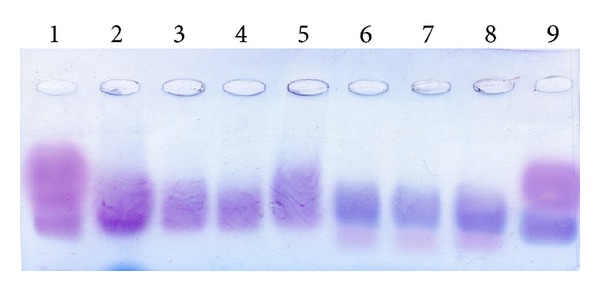
Foregram of ventral and dorsal aspects of GP of patient with III Grade Scheuermann's disease 1, 9: standard; 2: native GAG sample from the growth plate; 3, 4, and 5: GAG samples from the growth plate, annulus fibrosus, and nucleus pulposus, respectively, treated with keratanase; 6, 7, and 8: GAG samples from the growth plate, annulus fibrosus, and nucleus pulposus, respectively, treated with chondroitinase.

 This is in agreement with biochemical findings (Figure 4). 

**Figure 5:**
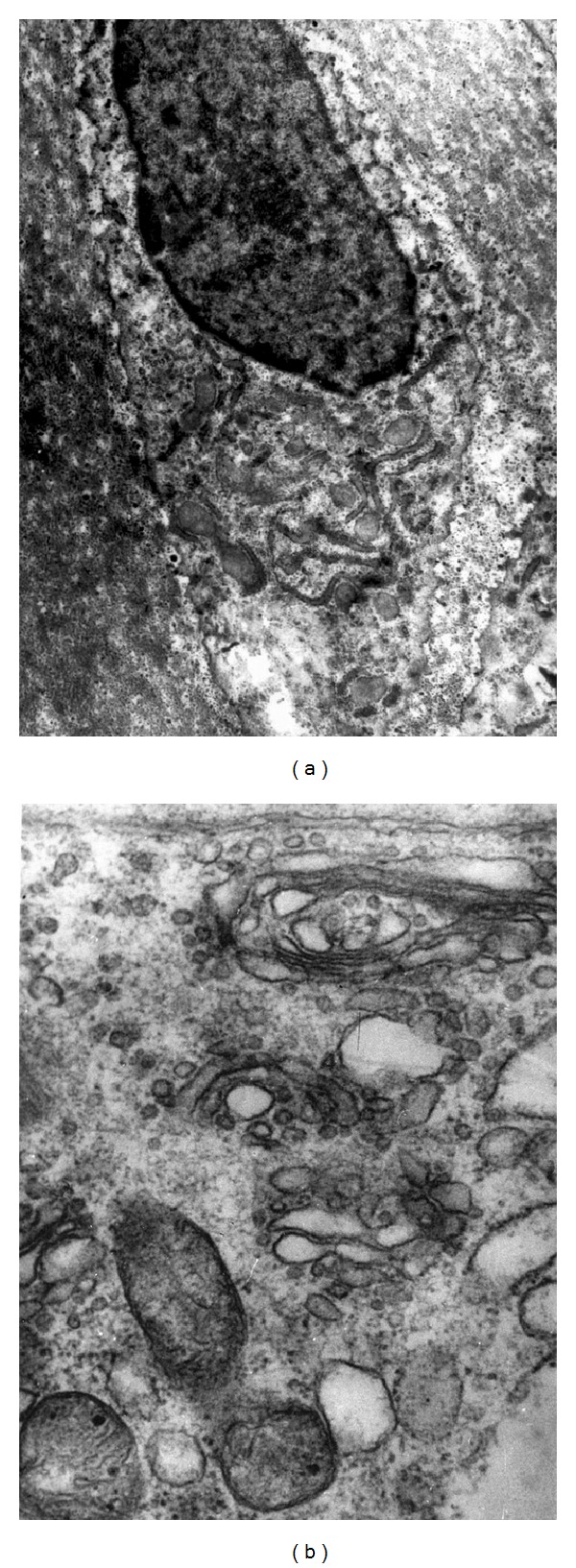
Ultrastructure of columnar chondrocytes in vertebral growth plate at the level of deformity: (a) ventral aspects; (b) dorsal aspects, ×5000.

*Ultrastructural arrangement *of chondrocytes in the ventral aspects is strongly modified (Figure 5(a)): isolated Golgi apparatus, inflated cisternae of the endoplasmic reticulum, and small mitochondria. Nucleus-free cells (ghost cells), more ultrastructurally survival cells too, occur in which, Golgi apparatus is located mainly near the nucleus, while its large vacuoles near membrane. The endoplasmic reticulum appears in these cells with a large number of attached ribosomes. Nuclei contain electron-dense chromatin assembly.

**Figure 6:**
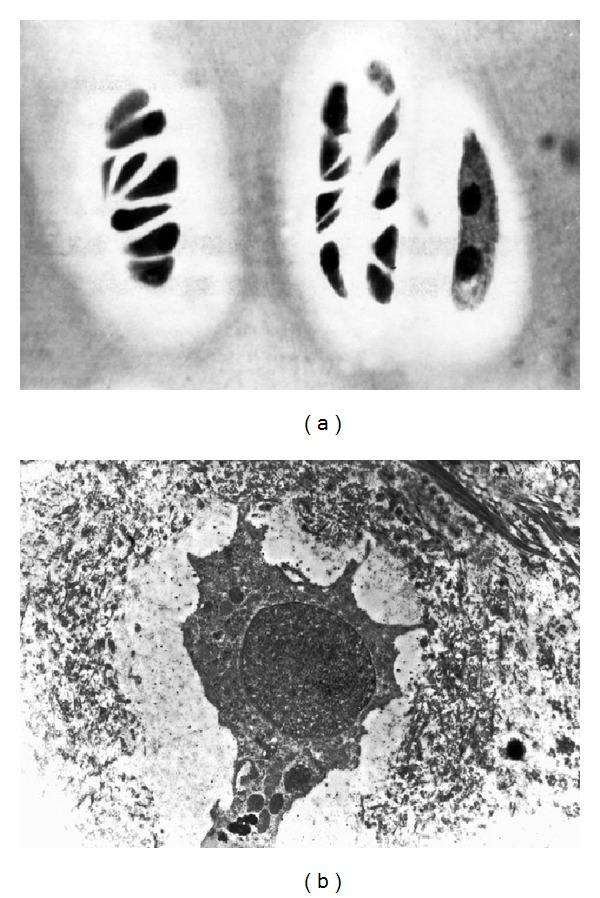
Barrier-trophic function of collagen-bound proteoglycans: (a) chondron's architectonics, columnar area of dorsal aspects. Semithin section. (b) Chondroblast is located in the center and surrounded by “free” and collagen-bound proteoglycans. Electron microscopy, ×5000.

Dorsal aspects are presented by cells of the columnar layer (Figure 5(b)) with off-center nuclei with homogeneous distribution of euchromatin. The Golgi apparatus with numerous vacuoles is distributed over the entire cytoplasmic surface. The endoplasmic reticulum contains attached ribosomes. Chondrocyte is surrounded by collagen-bound and diffuse proteoglycans (Figures 6(a) and 6(b)) forming a chondron structure which performs regulatory, trophic, and barrier functions, relations with hormonal system, and so forth. Ventral aspects do not have such chondron structure.

### 3.2. Biochemical Study of Ventral and Dorsal Aspects of the Vertebral Growth Plate

In the vertebral body growth plates, GAG counts lowered from 20.00 in ventral aspects to 3.00 *μ*g/mg in dorsal aspects. However, relative amount of keratan sulfate in ventral GP aspects reached 60% of the total GAG amount versus 17% in dorsal aspects. The level of sulfation was decreased in ventral aspects by a factor of four as compared with dorsal ones.

### 3.3. Conclusion

 Pathogenetic mechanism of spinal deformity development is a growth asymmetry underlaid by disorders in chondroblast proliferation and differentiation and in structural organization of the growth plate, as well as changes in the proteoglycan spectrum.

### 3.4. Candidate Gene Investigation

 Morphohistochemical and biochemical findings on proteoglycan spectrum alteration in ventral aspects of the growth plate—increase in keratin sulfate and decrease in chondroitin sulfate amount—gave a ground for investigation of the most representative proteoglycan gene in the growth plate—the aggrecan gene. It is known that aggrecan fulfills informational, barrier, and receptor functions and regulation of chondroblast differentiation and proliferation in the growth process [32]. Accretion of the detected keratin sulfate amount suggests a possibility of expression of the lumican gene containing three chains of keratin sulfate but no one of chondroitin sulfate. Indeed, isolated and cultivated chondroblasts of the growth plate from patients with Scheuermann's disease exhibited disorders in aggrecan expression but intact lumican expression [31]. Because of possible changes in gene expression in cultured cells every other study of gene expression was performed in noncultured chondroblasts isolated from vertebral growth plates (operational material) in accordance with above-mentioned techniques.

**Figure 7:**
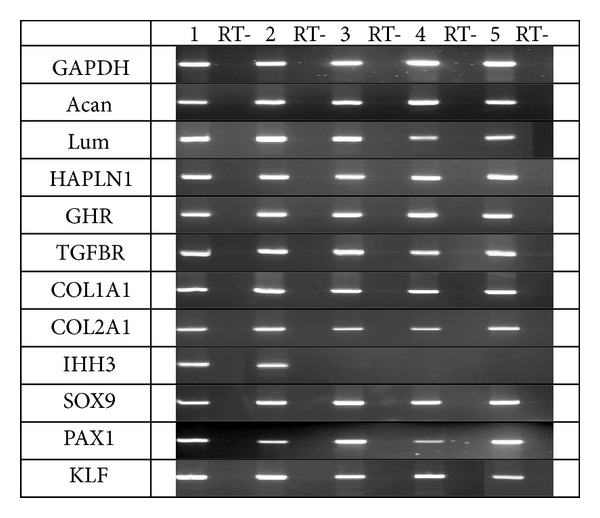
RT-PCR analysis of gene expression: 1: specimen from the growth plate of a healthy child; 2–5: specimens from growth plates of children with Scheuermann's disease; RT: negative control.

It was emerged that aggrecan gene, isoform 1 and isoform 2, and lumican are gene expressed both in ventral and dorsal aspects of the growth plate (morphological and biochemical findings (Figure 7)). If so, how can structural and functional distinctions between ventral and dorsal aspects be explained? A supposed disorder in expression of the link protein (HAPLN) as a part of aggrecan also did not prove true (Figure 7). 

A probability of disorder in chondroblast receptor function was not excluded. This supposition was based on the absence of monolayer in cultured chondroblasts from growth plates of Scheuermann's patients. We did not reveal any disorder in expression of gene receptor both to growth hormone and to transforming growth factor. Types 1 and 2 collagen genes were also expressed well.

Expression of the PAX1 gene typical only for fetal life or period of somite formation turned to be unusual. Expression of the IHH gene only in one studied case also remained unexplained, as well as proliferation gene expression in the absence of growth plate zonality. Proliferative activity is morphologically evident, though the process of columnar structure formation does not go on, but irregular proliferation is observed. Since transcription gene SOX9 is expressed in all cases, one can suppose that changes are caused by posttranslational disorders.

## 4. Conclusion

The paper summarizes longitudinal investigations of spinal deformities associated with Scheuermann's disease. Morphological and biochemical findings are presented selectively as possible markers of genetic pathology. Major gene dependency of the Scheuermann's disease was proved, and phenotypic criteria of inheritance were presented. Pathogenetic mechanisms of the disease development were identified: disorder in structural organization of ventral aspects of vertebral growth plates at the level of deformity, change in proteoglycan spectrum (keratin sulfate increase and chondroitin sulfate decrease). This study was followed up by identification of supposed candidate genes responsible for main structural transformations in a provisional cartilage. 

The study showed disorder in expression of IHH and PAX1 genes the latter is normally expressed only during embryogenesis. Expression of two aggrecan gene isoforms, link protein, receptors to growth hormone and growth factor, and types I and II collagen was preserved. High proliferation gene expression against the background of disordered proliferative activity of chondrocytes in the ventral aspects of the growth plate remains unexplained. Expression of the SOX9 gene or transcription gene may be the evidence of disorder in regulation at a posttranslational level. These findings are now verified. The paper objective was to share the obtained results with colleagues and propose joint investigations.

## Figures and Tables

**Table 1:** Primers and conditions for cartilage-related mesoderm marker RT-PCR.

Gene name	Primers name	Primer sequence	MgCl_2_ concentration, mM	Annealing temperature, °C	Target fragment size, bp
AGGRE	ACAN f ACAN r	5′-ATCGTCACCCCCGAGGAGCAG-3′ 5′-GGCGCTGGACAAACCCCTCTG-3′	3	60	379

LUM	LUM2 f LUM2 r	5′-GTGACTGGGCTGGGTCTCCCC-3′ 5′-GGCACTTGGGTAGCTTTCAGGGC-3′	4	58	329

HAPLN1	HAPLN1 f HAPLN1 r	5′-GGCAGAACACAGTGCCCGGAGTC-3′ 5′-GCGCACTGCAGCCTCAGTAGGAC-3′	4	58	317

GHR	GHR f GHR r	5′-GGCGAAGCTCGGAGGTCCTACA-3′ 5′-TGCATTGCGTGGTGCTTCCCATC-3′	4	58	568

TGFBR	TGFBR f TGFBR r	5′-GGCGAGCGGTCTTGCCCATC-3′ 5′-CTCAAGGCTTCACAGCTCTGCCA-3′	4	58	468

COL1A1	COL1A1 f COL1A1 r	5′-ATCCAGCTGACCTTCCTGCG-3′ 5′-TGGAAGCCGAATTCCTGGTCT-3′	4	58	301

COL2A1	COL2A1 f COL2A1 r	5′-GAAACCATCAATGGTGGCTTCC-3′ 5′-CGATAACAGTCTTGCCCCACTT-3′	4	58	322

IHH3	IHH3 f IHH3 r	5′-ACCACATCAGACCGCGACCGCAAT-3′ 5′-AGGCTGCACGTGGCTGGCAAATGT-3′	4	58	441

SOX9	SOX9 f SOX9 r	5′-GCGTCAACGGCTCCAGCAAGA-3′ 5′-GGCCTGCAGCGCCTTGAAGAT-3′	4	58	369

PAX1	PAX1 f PAX1 r	5′-ATCAGCCGCATCCTGCGCAACAA-3′ 5′-TCTCTAGCCCATTCACTGCGGGGT-3′	4	65	376

**Table 2:** Radiographic characteristics of Scheuermann's disease grades in probands.

Radiographic signs	Scheuermann's disease grade		
	I (*n* = 14)	II (*n* = 73)	III (*n* = 23)
Kyphosis (Cobb angle degrees)	25–45	45–65	>65
Number of segments involved	3-4	4-5	5–7

	Percentage of patients with a sign presence		

Endplate irregularities	65	100	68
Wedge-shaped vertebral body, >5 degrees	62	96	100
Schmorl's nodules	38	93	100
Vertebral body osteoporosis	31	64	68
Narrowing of intervertebral space	26	92	93
Apophysis fragmentation	19	69	52

**Table 3:** Distribution of relatives of probands with Scheuermann's disease depending on a kyphosis degree.

Relation degree	Total	Relatives with kyphosis (degrees)					
		25–45		45–65		>65	
		*n*	%	*n*	%	*n*	%
I–III	78 (100%)	41	52	31	40	6	8
I	41 (153%)	21	51	18	44	2	5
II	23 (29%)	14	61	6	26	3	13
III	14 (18%)	6	43	7	50	1	7

**Table 4:** Radiographic characteristics of relatives of probands with Scheuermann's disease.

Radiographic signs	Relation degree							
	I–III (*n* = 78)		I (*n* = 41)		II (*n* = 23)		III (*n* = 14)	
	*n*	%	*n*	%	*n*	%	*n*	%
Wedge-shaped vertebral body	69	89	38	93	15	65	13	97
Platyspondylia	9	12	2	56	8	35	—	—
Narrowing of intervertebral space	72	92	38	93	23	100	13	92
Vertebral body osteoporosis	63	81	33	80	23	100	9	66
Schmorl's nodules	33	42	15	37	—	12	13	93
Endplate irregulations	2	3	—	—	—	—	2	14
Apophysis fragmentation	—	—	—	—	—	—	—	—
Osteochondrosis signs	61	78	33	80	20	87	8	55
Spondylosis	44	56	25	61	7	30	9	66
Arthrosis	26	33	12	29	19	83	—	—

**Table 5:** Segregation analysis of Scheuermann's disease.

Genetic parameter	Classical Mendelian model	Dominant model		
		Obtained model of Mendelian transmission probabilities	Model of random transmission probabilities	Model of equal transmission probabilities
*q*	0.136	0.136 ± 0.037	0.134	0.421
*w*_*m*_ (*A*_1_ *A*_1_)	0.0	0.0	0.0	0.008
*w*_*m*_ (*A*_1_ *A*_2_)	1.0	1.0	1.0	0.981
*w*_*m*_ (*A*_2_ *A*_2_)	1.0	1.0	1.0	0.981
*w*_*f*_ (*A*_1_ *A*_1_)	0.0	0.0	0.0	0.073
*w*_*f*_ (*A*_1_ *A*_2_)	0.434	0.432 ± 0.065	0.433	0.321
*w*_*f*_ (*A*_2_ *A*_2_)	0.419	0.432	0.433	0.321
*qτ* (*A*_1_ *A*_1_)	1.0*	1.0*	1.0*	0.699
*qτ* (*A*_1_ *A*_2_)	0.5*	0.5*	0.496	0.699
*qτ* (*A*_2_ *A*_2_)	0.0*	0.0*	0	0.699
LH	160.285	LH_1_ = 160.286	LH_2_ = 160.285	LH_3_ = 170.834
AIC	334.570	330.572	336.570	353.668

*Parameter value is fixed.
